# Association of Anterior and Lateral Extraprostatic Extensions with Base-Positive Resection Margins in Prostate Cancer

**DOI:** 10.1371/journal.pone.0158922

**Published:** 2016-07-08

**Authors:** Yong Jin Kang, Mark Joseph Abalajon, Won Sik Jang, Jong Kyou Kwon, Cheol Yong Yoon, Joo Yong Lee, Kang Su Cho, Won Sik Ham, Young Deuk Choi

**Affiliations:** Department of Urology, Urological Science Institute, Yonsei University College of Medicine, Seoul, Republic of Korea; Taipei Medical University, TAIWAN

## Abstract

**Introduction:**

Positive surgical margins (PSM) detected in the radical prostatectomy specimen increase the risk of biochemical recurrence (BCR). Still, with formidable number of patients never experiencing BCR in their life, the reason for this inconsistency has been attributed to the artifacts and to the spontaneous regression of micrometastatic site. To investigate the origin of margin positive cancers, we have looked into the influence of extraprostatic extension location on the resection margin positive site and its implications on BCR risk.

**Materials & Methods:**

The clinical information and follow-up data of 612 patients who had extraprostatic extension and positive surgical margin at the time of robot assisted radical prostatectomy (RARP) in the single center between 2005 and 2014 were modeled using Fine and Gray’s competing risk regression analysis for BCR. Extraprostatic extensions were divided into categories according to location as apex, base, anterior, posterior, lateral, and posterolateral. Extraprostatic extensions were defined as presence of tumor beyond the borders of the gland in the posterior and posterolateral regions. Tumor admixed with periprostatic fat was additionally considered as having extraprostatic extension if capsule was vague in the anterior, apex, and base regions. Positive surgical margins were defined as the presence of tumor cells at the inked margin on the inspection under microscopy. Association of these classifications with the site of PSM was evaluated by Cohen’s Kappa analysis for concordance and logistic regression for the odds of apical and base PSMs.

**Results:**

Median follow-up duration was 36.5 months (interquartile range[IQR] 20.1–36.5). Apex involvement was found in 158 (25.8%) patients and base in 110 (18.0%) patients. PSMs generally were found to be associated with increased risk of BCR regardless of location, with BCR risk highest for base PSM (HR 1.94, 95% CI 1.40–2.68, p<0.001) after adjusting for age, initial prostate-specific antigen, pathologic Gleason score, and pathologic T stage in the multivariate model. Logistic regression for PSM site revealed no significant correlation of apex PSM with extraprostatic extension location, while base PSM was associated with increased odds of anterior (OR 2.513, 95% CI 1.425–4.430, p = 0.001) and lateral (OR 2.715, 95% CI 1.735–4.250, p<0.001) extraprostatic extension.

**Conclusion:**

Extension into the extraprostatic tissue on some specific locations do not share the same recur risk due to the different anatomical structures surrounding the organ. Anterior and lateral EPEs are prone to leave PSM on the base of the prostate, probably because of the lack of anatomical barricades slowing down the direct invasion process. More study on the pattern of spread of the tumors found to have extraprostatic extension is suggested for optimal planning of the operation extent and of the adjuvant radiotherapy.

## Introduction

Positive surgical margins have been presumed to be a finding related closely with the incomplete resection in the radical prostatectomy specimens, and have been regarded as an independent prognostic factor in predicting biochemical recurrence, with supporting evidences somewhat controversial.

While it has been shown in many circumstances that positive surgical margin (PSM) is associated with biochemical recurrence (BCR) risk, the fact that not all patients with PSM experience recurrence [1] remains not properly accounted for. According to Epstein et. al in their study, 6 out of 10 patients had no cancer in repeat biopsy. [2]

Retraction artifacts and lack of viable tumor cells due to cautery effect have been suggested as the possible causes [3, 4], but no data exists as of yet that clearly pinpoints the mechanism that lies behind the PSM without recurrence.

Authors of the current study focused on the possibility of the presence of anatomical barriers which blocks tumor growth to adjacent structures.

Extraprostatic extension have been considered as a prerequisite for PSM, except for the cases from capsular incision and artifact.[5] Topological association between extraprostatic extension and PSM has not well been documented. Areas with ambiguous capsules such as anterior wall, apex and base [6] may provide the tumor cells which achieved extraprostatic extension another ‘escape route’ to allow access to the lymphatic/vascular system.

To investigate the difference between PSM locations in terms of recurrence risk and to find their origin, BCR risk differences between the resection margin positive sites and the PSM odds between the extraprostatic extension locations have been compared.

## Materials and Methods

### Patient population

With approval from the Severance hospital institutional review board (protocol number 2015-2808-001), the clinical information and follow-up data of 1653 patients who underwent bilateral interfascial nerve-sparing robot assisted radical prostatectomy in the single center by the single operator (Y.D.C) between 2005 and 2014 were collected. Informed consent from the participants was waived by the institutional review board as the current study satisfied all of the following requirements for the waiver of informed consent:

- The research involved no more than minimal risk to the participants (retrospective data analysis of previously collected medical records)
- The waiver did not adversely affect the rights and welfare of the participants
- The research could not practicably be carried out without the waiver
- The participants were provided relevant information afterwards when necessary
- The research was not subject to MFDS-FDA regulation

Patients with extraprostatic extension at the time of operation (n = 884) were selected. Patients with missing data (n = 132), lymph node metastasis (n = 56), or extensive use of neoadjuvant androgen deprivation therapy (ADT) defined as prolonged duration (over 3 months) or maximal androgen blockade (MAB, n = 84) were excluded from the cohort, leaving 612 patients for analysis. Neoadjuvant hormone therapy in the study cohort consisted of Bicalutamide monotherapy, given majorly at patient’s request for pharmacotherapy before the operation.

### Pathologic examination

A single dedicated genitourinary pathologist (N.H.C) was responsible for the pathologic specimen analyses. Extraprostatic extensions were divided into categories according to location as anterior, lateral, posterolateral and posterior. Extraprostatic extensions were defined as presence of tumor beyond the borders of the gland in the posterior and posterolateral regions. Tumor admixed with periprostatic fat was additionally considered as having extraprostatic extension if capsule was vague in the anterior regions.

Positive surgical margins were defined as the presence of tumor cells at the inked margin on the inspection under microscopy. PSM locations were categorized into anterior, lateral, posterolateral, and posterior in match for extraprostatic extension. Apex and bladder neck margins were separately sent in search for positive margins.

### Postoperative follow-up

A patient was considered to have BCR when the postoperative prostate-specific antigen (PSA) level of 0.2 ng/mL above the nadir was detected after a nadir PSA value of 0.1 ng/mL was reached. Any need for additional radiotherapy, hormone therapy or chemotherapy was considered as BCR event.

### Statistical analysis

The cumulative incidence curve was plotted to calculate survival functions, and differences were assessed with the pairwise Gray’s test for equality with Bonferroni correction. Multivariate survival analysis was performed by constructing competing risk regression models. Assessment of correlation between EPE and PSM was done by calculating Cohen’s Kappa coefficient [7]. Odds ratios for categorical variables were calculated by Logistic regression. Plotting of cumulative incidence curve and analysis with Fine and Gray’s competing risk regression was performed using ‘cmprsk’ package in R (R version 3.2.1; R Foundation for Statistical Computing, Vienna, Austria). All other statistical analyses were performed using Statistical Package for Social Sciences v.22.0 for Windows (SPSS, Chicago, Illinois). A p-value < 0.05 was considered statistically significant in the current study.

## Results

### Baseline characteristics

Baseline characteristics have been listed in Table 1. Median follow-up duration was 36.5 months (IQR 20.1–36.5). PSM was found in 466 (76.1%) cases with extraprostatic extension. Apex involvement was found in 158 (25.8%) patients and base involvement in 110 (18.0%) patients. Of 213 (34.8%) patients received neoadjuvant ADT, 120 (56.3%) patients developed BCR, and in 157 (73.7%) patients PSM was identified. 276 (45.1%) patients have received adjuvant ADT after BCR was diagnosed.

**Table 1:** Baseline characteristics.

		Median (IQR)/n (%)
Age (at operation)	years	67 (61–71)
Initial PSA	ng/mL	9.7 (6.0–16.3)
Seminal vesicle invasion	(-)<pT3b	525 (85.8)
	(+): ≥pT3b	87 (14.2)
Invasion to other organs	(-): ≤pT3	595 (97.2)
	(+): pT4	17 (2.8)
Gleason score	≤6	94 (15.4)
	7	355 (58.0)
	8	63 (10.3)
	≥9	100 (16.3)
Neoadjuvant ADT	(-)	399 (65.2)
	(+)	213 (34.8)
Biochemical recurrence	(-)	319 (52.1)
	(+)	293 (47.9)
Adjuvant ADT	(-)	336 (54.9)
	(+)	276 (45.1)
EPE location	Anterior	88 (14.4)
	Lateral	266 (43.5)
	Posterolateral	33 (5.4)
	Posterior	282 (46.1)
PSM location	Apex	158 (25.8)
	Base	110 (18.0)
	Anterior	90 (14.7)
	Lateral	207 (33.8)
	Posterolateral	30 (4.9)
	Posterior	140 (22.9)

IQR: interquartile range;PSA: prostate-specific antigen;ADT: androgen deprivation therapy;EPE: extraprostatic extension;PSM: positive surgical margin.

### Time-to-event analysis

**Fig 1:**
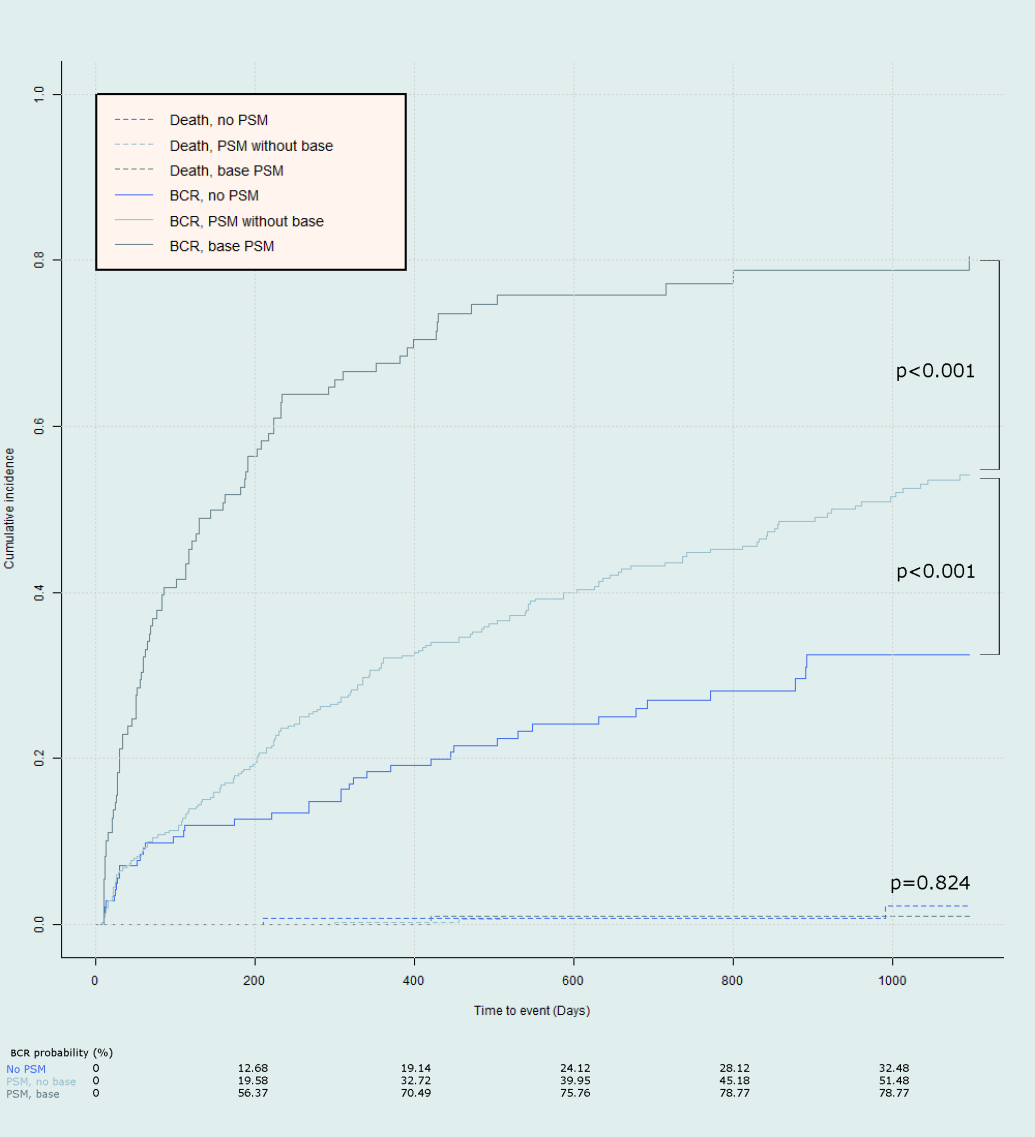
Cumulative incidence curve for BCR stratified according to PSM locations. A markedly increased rate of incidence is noted for the base PSM compared to other PSM locations (p<0.001, Gray’s test for equality). Deaths during follow-up have been plotted as separate dotted lines, which show no differences among groups (p = 0.824).

Patients were further categorized into groups with no PSM, PSM without positive base margin and PSM with base margin. Overall BCR was confirmed in 293 (47.9%) patients, with median follow-up period of 35.8 months (IQR 20.0–55.3). Total 12 (2.0%) of the patients have expired during the follow-up. 2 (0.3%), 6 (1.0%), and 4 (0.7%) of patients were from each group respectively with no statistically significant difference (p = 0.822). While PSM was associated with increased rate of BCR in margin positive sites other than base, the base margin positive patients experienced even higher rate of BCR, as shown in the comparison of cumulative incidence function from Fig 1 (Gray’s test for equality, p<0.001). From the cumulative incidence function, BCR probability during 3 year period for the PSM without base positive margin was 51.5% in contrast to 78.8% for the PSM with base positive margin.

### Multivariate survival analysis

On univariate analysis, initial PSA (≥20 ng/mL: p<0.001), Gleason sum (≥7: p<0.001), SVI (p<0.001), and neoadjuvant ADT (p = 0.010) were statistically significant predictors of BCR along with apex, base, anterior, lateral, and posterior PSM (p<0.001, p<0.001, p<0.001, p = 0.046, p<0.001, respectively), as shown in Table 2. Patient age (≥70 years: p = 0.710) and posterolateral PSM (p = 0.950) was not associated with increased risk of BCR in the analysis. With variables found significant in the univariate analysis included, multivariate analysis accounting for competing risk (follow-up loss due to death) was performed for the estimation of proportional subdistribution hazards.

**Table 2:** Competing risk regression analysis for biochemical recurrence, accounting for deaths during follow-up period.

	Univariate	Multivariate		
	*p-value*	HR^+^	95% CI	*p-value*
Age(≥70 years)	0.710	1.04	0.80–1.36	0.770
PSA(≥20 ng/mL)	<0.001*	1.85	1.37–2.49	<0.001*
Gleason score(≥7)	<0.001*	1.74	1.14–2.66	0.010*
Seminal vesicle invasion	<0.001*	1.97	1.42–2.74	<0.001*
Invasion to other organs	0.470	0.80	0.40–1.59	0.520
Neoadjuvant ADT	0.010*	1.15	0.89–1.48	0.280
PSM location				
Apex	<0.001*	1.56	1.20–2.03	<0.001*
Base	<0.001*	1.94	1.40–2.68	<0.001*
Anterior	<0.001*	1.32	0.92–1.88	0.130
Lateral	0.046*	1.34	1.02–1.75	0.034*
Posterolateral	0.950			
Posterior	<0.001*	1.79	1.35–2.36	<0.001*

HR: hazard ratio;CI: confidence interval;PSA: prostate-specific antigen;ADT: androgen deprivation therapy;PSM: positive surgical margin

*statistically significant at p<0.05

^+^hazard ratios displayed are subdistribution hazards accounting for competing risks due to death.

Age, invasion to adjacent organs, neoadjuvant ADT and anterior (p = 0.770, 0.520, 0.280, and 0.130, respectively) PSMs were not significantly associated with increased risk of BCR. BCR risk was highest for base PSM (HR 1.94, 95% CI 1.40–2.68, p<0.001) after adjusting for age, initial prostate-specific antigen, pathologic Gleason score, seminal vesicle invasion, invasion to other organs, and use of neoadjuvant ADT in the multivariate competing risk regression model. Apex (HR 1.56, 95% CI 1.20–2.03, p<0.001) and posterior (HR 1.79, 95% CI 1.35–2.36, p<0.001) PSM showed increased risk of BCR as well.

### Association between PSM and EPE site

To test for concordance of EPE in PSM sites, Cohen’s Kappa was calculated to measure the degree of agreement of each EPE-PSM pair for their location (Table 3). Concordant occurrence of PSM and EPE was observed, as the pairs generally showed weak positive correlation. Weak to moderate correlation in anterior and lateral tumors was noted (0.540, and 0.634, respectively). Despite the positive correlation, considerable discordance was expected as the coefficient of determination in anterior, posterolateral, and posterior tumors were low (0.292, 0.232, and 0.138, respectively), showing that the substantial amount of data are not explained by the correlation.

**Table 3:** Correlation between EPE location and PSM sites.

PSM and EPE location	Correlation coefficient^+^	Coefficient of determination^¶^	*p-value*
Anterior	0.540	0.292	<0.001*
Lateral	0.634	0.402	<0.001*
Posterolateral	0.482	0.232	<0.001*
Posterior	0.372	0.138	<0.001*

PSM: positive surgical margin;EPE: extraprostatic extension

*statistically significant at p<0.05

^+^Cohen’s Kappa(к)

^¶^Calculated as к^2^.

Logistic regression for PSM site revealed no significant association of apex PSM with extraprostatic extension location, with exception of posterior side where odds decreased (OR 0.574, 95% CI 0.395–0.834, p = 0.004), while base PSM was associated with increased odds of anterior (OR 2.513 95% CI 1.425–4.430 p = 0.001) and lateral (OR 2.715, 95% CI 1.735–4.250, p<0.001) extraprostatic extension as shown in Table 4. Effect of neoadjuvant ADT on PSM was not statistically significant (p = 0.302).

**Table 4:** Logistic regression for apex and base PSM. Note higher odds of anterior, lateral EPE for the base PSM. Effect of neoadjuvant ADT on PSM was not statistically significant.

PSM location	EPE location/NHT	Univariate	Multivariate		
		*p-value*	OR	95% CI	*p-value*
Apex	Anterior	0.736			
	Lateral	0.004*			0.277
	Posterolateral	0.844			
	Posterior	0.036*	0.574	0.395–0.834	0.004*
Base	Anterior	0.031*	2.513	1.425–4.430	0.001*
	Lateral	<0.001*	2.715	1.735–4.250	<0.001*
	Posterolateral	0.368			
	Posterior	0.002*			0.775
Posterior	Posterior	<0.001*	10.473	6.464–16.967	<0.001*
Any	Neoadjuvant ADT	0.302			

PSM: positive surgical margin;EPE: extraprostatic extension;NHT: neoadjuvant hormone therapy;OR: odds ratio;CI: confidence interval; PSA: prostate-specific antigen; ADT: androgen deprivation therapy

*statistically significant at p<0.05.

## Discussion

### Unique anatomy of the anterior extraprostatic space

Anatomy of the anterior extraprostatic space which spans across apex through base, is unique in that capsule is vaguely defined and is covered with fibromuscular shielding (anterior fibromuscular stroma: AFMS).[8] Not only lack of capsular structure makes it difficult to define EPE and PSM in these regions, intertwining of AFMS with surrounding muscular structures may provide alternative route through which malignant cells can spread and gain access to the lymphatic drainage system. We have assumed the preference of prostate cancer invasion process to be nonequivalent throughout the extraprostatic space, based on their anatomical differences.

### Differences in BCR risk according to PSM site

Result from the current study demonstrates that each PSM sites show diverse degree of association with EPE locations, with varying BCR risk. In our data, base PSM (HR 1.94, 95% CI 1.40–2.68, p<0.001) showed higher risk of BCR when adjusted for known prognostic factors, including Gleason sum ≥7 (HR 1.74, 95% CI 1.14–2.66, p = 0.010) whereas anterior PSM (p = 0.130) was of less value when predicting BCR risk.

There have been previous reports describing differences in disease recurrence risk according to the anatomical orientation of EPE and PSM. Anterior prostate cancers are reported to be associated with lower rate of EPE while PSM rate was higher compared to posterior cancers. According to Koppie et al., although specimens with posterior prostate cancers showed higher rates of extraprostatic extension, anterior prostate cancers had higher tumor volume and PSM rate than those with their posterior counterparts. They have pointed out that the anterior prostate includes the anterior horns of the peripheral zone and the AFMS and the clinical behavior of tumors originating from the anterior peripheral zone is largely unknown. They have also noted that anterior prostate cancers were more likely than posterior prostate cancers to have PSMs at the bladder neck [9], which is supported by the results demonstrated in the current study where anterior EPE was closely associated with base PSM (OR 2.513 95% CI 1.426–4.430 p = 0.001)

Positive margin found in the bladder neck (prostate base) has been known to accompany relatively higher recur risk compared to other locations. However, Bladder neck margins have been a source of continuous debate whether it should be considered as T4 disease until the recent AJCC stage modification. [10, 11]

Data from the current study is in line with previous studies where microscopic bladder neck involvement shows association with increased recur risk, although the association was not as strong as invasion of seminal vesicle or other organs. [12–14]

Considering the diverse degree of association the posterior (HR 1.79, 95% CI 1.35–2.36, p<0.001) and apex (HR 1.56, 95% CI 1.20–2.03, p<0.001) PSM showed with increased BCR risk, variability in recur risk may exist, which can play as a contributing factor behind inconsistent BCR rate in PSM patients.

Our results differed from the study conducted by Eastham et al. where posterolateral PSM was brought up to be the major source of BCR.[15] Profound shift towards incidence of posterolateral PSM was noted in the study. As detection of PSM is often disrupted by effects from cautery, tear artifacts, Stephenson et al. have suggested that this may be the reason behind such results as intraoperative trauma to the specimen is least likely to occur at this location.[1] Although their results were different from ours in that they concluded the location of PSM makes little difference in predicting BCR risk, our data enables more detailed comparison between PSM locations, with PSM site other than apex also subcategorized for analysis.

### Discordance in EPE and PSM locations

Considering the prevalent discordance between EPE and PSM sites reported in several studies, it is questionable whether EPE should be present at the PSM site as a prerequisite. In radical prostatectomy specimens, EPE is known to occur most commonly in posterolateral mid-prostate. According to previous literatures, extraprostatic extension is most frequently encountered in the posterolateral region at the neurovascular bundle.[16] In a study conducted for the RARP patients with concurrent EPE and PSM also revealed that EPE occurred most commonly in the posterolateral mid-prostate. [6]

In comparison to EPE, apex and base (especially anterior) have been reported as the most common site for PSM along with posterolateral side. In their multi-centered study involving 8418 RARP patients, Patel et al. found the incidence of apex PSM to be the highest (36%), followed by posterior PSM (29%). [17, 18] Regarding apex PSM, there is a possibility of artifact from the lack of discernable capsule. This view is supported by previous literatures as many apical PSMs did not confer an increased risk of BCR. [1,19,20]

In their search for an association between BCR risk and EPE/PSM site discordance, Johnson et al. reported that in contrast to EPE frequently occurring in the mid-gland region, PSM was most frequent at the base, specifically the anterior base. Overall 51.8% of the cases had EPE and PSM in discordant locations, 19.6% had EPE and PSM in the same location, and 28.6% had areas of EPE and PSM both in the same location as well as in different locations [6] In our data, inconsistency between EPE and PSM sites was also noted, as of all 467 patients with PSM, 157 PSMs (33.6%) occurred at sites where EPE was not present. This high rate of discordance between EPE and base PSM remains not properly accounted for. The weak yet positive correlation of EPE with PSM site (0.540, 0.634, 0.482, 0.372 for anterior, lateral, posterolateral, and posterior tumors, respectively) leaves the room for the existence of PSMs whose origins are not properly explained.

Data concerning the concordance between EPE and PSM and its impact upon BCR risk is scarce. In general, simultaneous EPE and PSM presence alerts worse prognosis as shown in multiple studies. In T3a/b patients, patients with positive surgical margins are reported to have a BCR free survival rate that is significantly lower according to Kausik et al.[21]. Similar conclusions were drawn from the study by Cheng et al., where 5-year progression free survival was 78% for those with positive margins alone, 55% for those with both positive margins and EPE, and 90% for those with negative margins and EPE.[22]

There are reports on PSMs that occurs without coexisting EPE, such as a result of capsular incision in T2 disease. In T3a patients, risk of BCR is reported to be same or less compared to PSM without capsular incision, lying somewhere between pT3a margin negative and pT3a margin positive patients. Capsular incision has been defined as tumor extending to the inked margin with the absence of histologically documented EPE and the prognostic significance of capsular incision was similar to that of pT3a with positive margins. [23] Similar conclusions were drawn from the study by Barocas et al. which compared the early recurrence risk of capsular incision into tumor and into benign glands, rendering capsular incision not statistically different from other pathologic findings.[24]

Also in tumors with both EPE and PSM, concordant and discordant EPE-PSM location show similar risk of BCR. Johnson et al. pointed out that while EPE and PSM may occur in the same location, they often did not. The lack of overlap of EPE and PSM found in many cases in this cohort suggests that at least some PSMs are influenced by more than disease pT classification [6] In the 696 patients with T3 or more advanced stage and PSMs, Stephenson et. al revealed that PSMs at a site of EPE were associated with a similar risk of BCR compared to those at a site where there was no capsular penetration (7-year progression free survival 40% vs 42%, p = 0.2).[1]

With no significant difference in terms of BCR whether PSM originates from capsular incision or from the EPE in close proximity, exceptionally higher recur risk associated with concurrent EPE and PSM mandates the search for other factor in action that needs consideration.

### Association of anterior/lateral EPE tumors with base PSM

The anatomical structure of the anterior-lateral side may have a vulnerability which leads to base margin positivity. In our data, all PSMs other than those of the apex and base were correlated with EPE in concordant locations. In contrast to these PSMs, which may have resulted from capsular incision or unresected EPE, base PSM showed statistically significant association with anterior (OR 2.513, 95% CI 1.425–4.430, p = 0.001) and lateral EPE (OR 2.715, 95% CI 1.735–4.250, p<0.001), suggesting an alternative etiology.

Anterior side of prostate is covered by coarse layer of fibromuscular tissue before reaching extraprostatic fat. The AFMS is reported to be variably intertwined with skeletal muscle fibers of the urogenital diaphragm (apex) and levator ani (mid) muscles, or fuse with smooth muscle of the detrusor (base), and contains blood vessels that supply/drain the anterior prostate (apex through base). [8] While it is widely accepted that prostate cancers mainly arise from peripheral zone, and transitional zone cancers show association with less aggressive features, anterior side lies in close proximity to the anterior horn of peripheral zone, leaving the site vulnerable to direct invasion. [8] Possibility of extraprostatic route of spread should be thoroughly investigated. Fine et. al postulated that this region may represent a primary site for expansile growth of anterior tumors irrespective of zone of origin.[8]

Although prostate cancers show tropism toward ipsilateral lymph nodes, formidable number of patients are still found to bear metastatic lymph node on the contralateral side. McLaughlin et. al previously demonstrated that metastatic lymph node is frequently bilateral (57%) when present [25]. According to Weckermann et al., in unilateral disease, four of five LN-positive men had LN metastases on the same side of the pelvis and one had positive LNs bilaterally. [26]

While there seems to be no other communication between contralateral lymph nodes, finding of bilateral lymph node involvement in unilateral disease suggests the possibility for AFMS to serve as a bridge between two lobes.

### Posterior EPE tumors tend to remain confined in comparison to others

Higher tendency of posterior EPE to remain confined in comparison to the anterior and lateral EPE was noted in the logistic regression with apex PSM specimens (OR 0.574, 95%CI 0.395–0.834, p = 0.004). Posterior prostate is relatively well-protected by nearby fascia structures. Pelvic cavity enclosing the prostate is covered by fasciae, some forming compartmented space by fusing into each other. Fasciae surrounding the extraprostatic space may form a ‘trap’ for the tumors extending outside the posterior prostatic capsule, slowing down the direct invasion process.

Previous review by Walz et al. provides good overview on the fasciae enclosing the posterior prostate.[27] Posteriorly lies the multilayered Denonvillier’s fascia dividing the space between the rectum and the prostatic fascia. The fasica is very often fused with the prostate capsule at the center of the posterior prostatic surface [27], forming additional compartment in the posterior side. The space is delineated anteriorly by prostatic fascia which encapsulates prostate. Between the posterior bladder and prostate, vesicoprostatic muscle forms a thin layer which consists of smooth muscle [28]. Endopelvic fascia, which covers levator ani medially, forms the lateral wall of the compartment fusing with AFMS at the anterior prostate [29]. Denonvillier’s fascia extends distally to the apex of the prostate to end at the prostatourethral junction in a terminal plate in continuity with the central perineal tendon, covering the distal end of the compartment. [27]

Peripheral zone prostate cancers are renowned for tropism for the perineural tissues, and it has been postulated that most of the posterior cancers commonly achieve EPE via neurovascular bundle located near the posterolateral pedicle of the prostate.

According to 2011 ISUP consensus, extraprostatic extension from peripheral zone cancers is most commonly identified in the posterolateral aspect of the gland making the tracking of the tumor along the neurovascular bundle most common mechanism of spread. [30] This holds true in the study by Koppie et al. as the most common locations of PSMs for posterior cancers were the posterior apex (36%), posterior mid (25%) and apical section (24%) [9], with posterior PSMs occurring more in the posterior cancers.

However, literatures assessing oncological outcomes for the nerve sparing technique reveals that recur risks were not significantly different from the cases where the wide excision was done [31]. This indirectly indicates that NVB involvement is not a factor that significantly compromises recur risk. Rather, tumor extension beyond the compartment formed by surrounding structures may stand as an major hurdle before metastasizing into lymph nodes/distant organs, considering the exclusively high odds of posterior EPE in posterior PSM (OR 10.473, 95% CI 6.464–16.967, p<0.001) patients from the current study, with no association found for other EPE sites.

### Limitations

The current study was limited by the high rate of patients who had previously undergone neoadjuvant ADT (35%), which is known to affect the rate of PSM. We attempted to minimize the effect by not including patients with extensive exposure in the cohort; however, potential aberration could not be excluded. Although not statistically significant in our multivariate model for BCR, the association on univariate analysis suggests a possible overlap of patients with high-risk factors for BCR (higher T stage, PSA, or Gleason score) and patients who received neoadjuvant ADT. While no specific patient group was selected to receive ADT, elimination of the possible effect of ADT on BCR may have improved the quality of the analysis.

While our data point to a possible alternative route represented by AFMS, we were unable to exclude other possibilities, such as other routes of metastasis. As no data concerning nodal status or distant metastasis were available in the current study, an additional investigation is required to properly assess the impact of invasion via extraprostatic tissues on the base PSM.

The overall rate of PSM in EPE patients was 76.2%, with 54.3% of patients with PSM experiencing BCR. This rate exceeds the higher end of the previously reported BCR rate of 5%–27%. This markedly high rate may partly be explained by the higher proportion of patients with a Gleason sum above 7 in the current study (84.6%), hence increasing the possibility of presenting with more advanced disease at the time of operation. To generalize our findings to a more general population beyond our institute, future studies with a larger population that compare data with other centers with different settings are necessary.

## Conclusion

It seems orientation of invasion into the extraprostatic space does not bear homogeneous weight in the clinical picture. Risk of metastasis was significantly higher in the base margin positive cancers. PSM should be separately considered according to the location.

Extended tumors into the extraprostatic tissue on some specific locations do not share the preference in the direction of spread due to the different anatomical structures surrounding the organ. Especially anterior and lateral EPEs are prone to result in PSM on the base of the prostate, probably related to the lack of capsule on the anterior surface, while posterior space is relatively conserved due to multilayered anatomical barricades slowing down the direct invasion process.

AFMS and anterior extraprostatic space stands as a potential route of spread in patients with anterior/lateral extraprostatic extension. Further study regarding the pattern of spread for the anterior tumor is suggested for optimal planning of the operation extent and of the adjuvant radiotherapy.

## Supporting Information

S1 File Raw data of study cohort. (XLSX)

## References

[1] Stephenson AJ, Wood DP, Kattan MW, Klein EA, Scardino PT, Eastham JA, et al Location, extent and number of positive surgical margins do not improve accuracy of predicting prostate cancer recurrence after radical prostatectomy. J Urol. 2009;182(4):1357–63. 10.1016/j.juro.2009.06.046 19683274

[2] Epstein JI. Evaluation of radical prostatectomy capsular margins of resection. The significance of margins designated as negative, closely approaching, and positive. Am J Surg Pathol. 1990;14(7):626–32. 2356922 10.1097/00000478-199007000-00003

[3] Marks RA, Koch MO, Lopez-Beltran A, Montironi R, Juliar BE, Cheng L. The relationship between the extent of surgical margin positivity and prostate specific antigen recurrence in radical prostatectomy specimens. Hum Pathol. 2007;38(8):1207–11. 17490720 10.1016/j.humpath.2007.01.006

[4] Evans AJ, Henry PC, Van der Kwast TH, Tkachuk DC, Watson K, Lockwood GA, et al Interobserver variability between expert urologic pathologists for extraprostatic extension and surgical margin status in radical prostatectomy specimens. Am J Surg Pathol. 2008;32(10):1503–12. 10.1097/PAS.0b013e31817fb3a0 18708939

[5] Psutka SP, Feldman AS, Rodin D, Olumi AF, Wu CL, McDougal WS. Men with organ-confined prostate cancer and positive surgical margins develop biochemical failure at a similar rate to men with extracapsular extension. Urology. 2011;78(1):121–5. 10.1016/j.urology.2010.10.036 21411125

[6] Johnson MT, Ramsey ML, Ebel JJ, Abaza R, Zynger DL. Do robotic prostatectomy positive surgical margins occur in the same location as extraprostatic extension? World J Urol. 2014;32(3):761–7. 10.1007/s00345-013-1149-5 24096432

[7] Marry L. McHugh. Interrater reliability: the kappa statistic. Biochemia Medica 2012;22(3):276–82. 23092060 PMC3900052

[8] Fine SW, Al-Ahmadie HA, Gopalan A, Tickoo SK, Scardino PT, Reuter VE. Anatomy of the anterior prostate and extraprostatic space: a contemporary surgical pathology analysis. Adv Anat Pathol. 2007;14(6):401–7. 18049129 10.1097/PAP.0b013e3181597a9c

[9] Koppie TM, Bianco FJ Jr., Kuroiwa K, Reuter VE, Guillonneau B, Eastham JA, et al The clinical features of anterior prostate cancers. BJU Int. 2006;98(6):1167–71. 17026586 10.1111/j.1464-410X.2006.06578.x PMC2239295

[10] Dash A, Sanda MG, Yu M, Taylor JM, Fecko A, Rubin MA. Prostate cancer involving the bladder neck: recurrence-free survival and implications for AJCC staging modification. American Joint Committee on Cancer. Urology. 2002;60(2):276–80. 12137826 10.1016/s0090-4295(02)01727-2

[11] Yossepowitch O, Engelstein D, Konichezky M, Sella A, Livne PM, Baniel J. Bladder neck involvement at radical prostatectomy: positive margins or advanced T4 disease? Urology. 2000;56(3):448–52. 10962313 10.1016/s0090-4295(00)00676-2

[12] Pierorazio PM, Epstein JI, Humphreys E, Han M, Walsh PC, Partin AW. The significance of a positive bladder neck margin after radical prostatectomy: the American Joint Committee on Cancer Pathological Stage T4 designation is not warranted. J Urol. 2010;183(1):151–7. 10.1016/j.juro.2009.08.138 19914651

[13] Aydin H, Tsuzuki T, Hernandez D, Walsh PC, Partin AW, Epstein JI. Positive proximal (bladder neck) margin at radical prostatectomy confers greater risk of biochemical progression. Urology. 2004;64(3):551–5. 15351591 10.1016/j.urology.2004.04.003

[14] Poulos CK, Koch MO, Eble JN, Daggy JK, Cheng L. Bladder neck invasion is an independent predictor of prostate-specific antigen recurrence. Cancer. 2004;101(7):1563–8. 15378493 10.1002/cncr.20551

[15] Eastham JA, Kuroiwa K, Ohori M, Serio AM, Gorbonos A, Maru N, et al Prognostic significance of location of positive margins in radical prostatectomy specimens. Urology. 2007;70(5):965–9. 18068455 10.1016/j.urology.2007.08.040

[16] Egevad L, Srigley JR, Delahunt B. International Society of Urological Pathology (ISUP) consensus conference on handling and staging of radical prostatectomy specimens: rationale and organization. Mod Pathol. 2011;24(1):1–5. 10.1038/modpathol.2010.159 20802466

[17] Coelho RF, Chauhan S, Orvieto MA, Palmer KJ, Rocco B, Patel VR. Predictive factors for positive surgical margins and their locations after robot-assisted laparoscopic radical prostatectomy. Eur Urol. 2010;57(6):1022–9. 10.1016/j.eururo.2010.01.040 20163911

[18] Patel VR, Coelho RF, Rocco B, Orvieto M, Sivaraman A, Palmer KJ, et al Positive surgical margins after robotic assisted radical prostatectomy: a multi-institutional study. J Urol. 2011;186(2):511–6. 10.1016/j.juro.2011.03.112 21680001

[19] Blute ML, Bostwick DG, Bergstralh EJ, Slezak JM, Martin SK, Amling CL, et al Anatomic site-specific positive margins in organ-confined prostate cancer and its impact on outcome after radical prostatectomy. Urology. 1997;50(5):733–9. 9372884 10.1016/S0090-4295(97)00450-0

[20] Fesseha T, Sakr W, Grignon D, Banerjee M, Wood DP Jr., Pontes JE. Prognostic implications of a positive apical margin in radical prostatectomy specimens. J Urol. 1997;158(6):2176–9. 9366338 10.1016/s0022-5347(01)68189-8

[21] Kausik SJ, Blute ML, Sebo TJ, Leibovich BC, Bergstralh EJ, Slezak J, et al Prognostic significance of positive surgical margins in patients with extraprostatic carcinoma after radical prostatectomy. Cancer. 2002;95(6):1215–9. 12216087 10.1002/cncr.10871

[22] Cheng L, Darson MF, Bergstralh EJ, Slezak J, Myers RP, Bostwick DG. Correlation of margin status and extraprostatic extension with progression of prostate carcinoma. Cancer. 1999;86(9):1775–82. 10547551 10.1002/(sici)1097-0142(19991101)86:9<1775::aid-cncr20>3.0.co;2-l

[23] Shuford MD, Cookson MS, Chang SS, Shintani AK, Tsiatis A, Smith JA Jr., et al Adverse prognostic significance of capsular incision with radical retropubic prostatectomy. J Urol. 2004;172(1):119–23. 15201750 10.1097/01.ju.0000132137.02846.ec

[24] Barocas DA, Han M, Epstein JI, Chan DY, Trock BJ, Walsh PC, et al Does capsular incision at radical retropubic prostatectomy affect disease-free survival in otherwise organ-confined prostate cancer? Urology. 2001;58(5):746–51. 11711353 10.1016/s0090-4295(01)01336-x

[25] McLaughlin AP, Saltzstein SL, McCullough DL, Gittes RF. Prostatic carcinoma: incidence and location of unsuspected lymphatic metastases. J Urol. 1976;115(1):89–94. 1246119 10.1016/s0022-5347(17)59078-3

[26] Weckermann D, Holl G, Dorn R, Wagner T, Harzmann R. Reliability of preoperative diagnostics and location of lymph node metastases in presumed unilateral prostate cancer. BJU Int. 2007;99(5):1036–40. 17437437 10.1111/j.1464-410X.2007.06791.x

[27] Walz J, Burnett AL, Costello AJ, Eastham JA, Graefen M, Guillonneau B, et al A critical analysis of the current knowledge of surgical anatomy related to optimization of cancer control and preservation of continence and erection in candidates for radical prostatectomy. Eur Urol. 2010;57(2):179–92. 10.1016/j.eururo.2009.11.009 19931974

[28] Secin FP, Karanikolas N, Gopalan A, Bianco FJ, Shayegan B, Touijer K, et al The anterior layer of Denonvilliers' fascia: a common misconception in the laparoscopic prostatectomy literature. J Urol. 2007;177(2):521–5. 17222624 10.1016/j.juro.2006.09.028

[29] Kiyoshima K, Yokomizo A, Yoshida T, Tomita K, Yonemasu H, Nakamura M, et al Anatomical features of periprostatic tissue and its surroundings: a histological analysis of 79 radical retropubic prostatectomy specimens. Jpn J Clin Oncol. 2004;34(8):463–8. 15371464 10.1093/jjco/hyh078

[30] Magi-Galluzzi C, Evans AJ, Delahunt B, Epstein JI, Griffiths DF, van der Kwast TH, et al International Society of Urological Pathology (ISUP) Consensus Conference on Handling and Staging of Radical Prostatectomy Specimens. Working group 3: extraprostatic extension, lymphovascular invasion and locally advanced disease. Mod Pathol. 2011;24(1):26–38. 10.1038/modpathol.2010.158 20802467

[31] Sofer M, Hamilton-Nelson KL, Schlesselman JJ, Soloway MS. Risk of positive margins and biochemical recurrence in relation to nerve-sparing radical prostatectomy. J Clin Oncol. 2002;20(7):1853–8. 11919244 10.1200/JCO.2002.07.069

